# Mortality of Philadelphia, for April, May, and June, 1857

**Published:** 1857-11

**Authors:** Wilson Jewell


ART. VIII.—*Mortality of Philadelphia, for April, May, and June*, 1857, *collated from the Health Office Record.* By WILSON JEWELL, M.D.

THE deaths for the second quarter of the year, from March 28th to June 27th, embracing a period of 92 days, have amounted to 2398. This statement shows a comparatively healthy state of our city for the past three months. The number of deaths having been 11 per cent. less than the previous quarter, and 3 per cent. less than the second quarter of 1856.

The deaths recorded from disease, number 1971, while those charged to adventitious causes, among which we have placed stillborn and unknown, amount to 427. By this classification, we show a mortality of not more than 21½ per day, from disease, or, for the quarter, as one in every 279 of the population, estimating this at 550,000.

The deaths among males have been 1298, and among females 1100, presenting an unusual excess in the male sex of 8·21 per cent.

The mortality in children, or those under twenty years, has amounted to 1329; exceeding those over 20 by 260, or 10 per cent. The deaths among boys were 716, and among girls 613; being an excess of more than 7 per cent. of the former.

The male stillborn children exceeded those of the female sex by 20 per cent., an unusually large percentage, but whether accidentally, or the result of natural causes, remains to be shown hereafter.

Among children under five years of age, there are recorded 941 deaths, not including the stillborn, which have been 125. This mortality is less than usual, amounting only to 41 per cent. Those under one year, were 19 per cent.

The month of June has been remarkably healthy; according to the record, only 635 deaths have been registered. The last week in this month gave only 131 deaths. This was the lowest during the quarter. The highest number for any one week, was in that from the 11th to the 18th of April, when they reached 226.

The deaths from "zymotic" diseases, have fallen off nearly one half those of the previous quarter. This is owing, in a great degree, to the reduced number of deaths from scarlet fever, equal to 272 for the quarter. Besides this, in consequence of the absence of the usual temperature of June, there are only 10 deaths from cholera infantum. Last year, in the same quarter, there were 32 deaths recorded. A great disproportion may also be noticed between the deaths from small-pox last year, in the second quarter, and those in the present, equal to 58 per cent.

Scarlet fever is still lingering in our city, although the cases as well as deaths have materially diminished since our published report for the first quarter of the year.

The "Sporadic, or Uncertain" class of diseases, make up 338 of the deaths,—the same number as recorded in the parallel quarter of last year. Nearly one half of the deaths registered under this head, are from debility and marasmus. Dropsy (what description?) furnishes 57, and nearly one-half of these were among children.

Of the class "Nervous" diseases, there were 397 deaths, and of this number, 264, or 20·12 per cent., were under twenty years of age. Convulsions, principally in infants, or those under two years, contributes 121 of the total. Inflammation of the brain gives 62, and congestion of the brain 44. All of these were among children.

The diseases of the "Organs of Respiration" furnish a larger proportion of deaths than any other class, amounting to 661. Of these, consumption of the lungs contributes 359, equal to 52·64 per cent., and 15·78 per cent. of the whole number of deaths (excepting stillborn). The deaths under this class, from inflammation of the respiratory organs, amounted to 220, or an increase of 14 per cent. above those for the same quarter of last year. The deaths from consumption of the lungs, however, were less than for the same period in 1856.

Under the name "Disease of the Heart" (what kind?), we find 47 deaths recorded. Inflammation of the stomach and bowels, 65 deaths. Puerperal fever, 12 deaths. This last is an improvement over those for the last quarter; nearly one-third less.

The deaths from "External Causes" amount to 104. Of these, 44, or 40·54 per cent., were under the head, drowned, and 19 were ascribed to casualties.

TABLE No. 1.

*Deaths for the Second Quarter of the Year* 1857, *classified.*

| | April. | May | Jun | Male. A. | Male. M. | Male. J. | Female A. | Female M. | Female J. | Total. |
|---|---|---|---|---|---|---|---|---|---|---|
| 1. *Endemic and Contagious Diseases.* Zymotic or Epidemic, . . . . . | 193 | 113 | 99 | 89 | 62 | 41 | 104 | 51 | 58 | 405 |
| 2. *Uncertain or General Seat.* Sporadic Diseases, . . . . . . | 130 | 108 | 100 | 72 | 60 | 44 | 58 | 48 | 56 | 338 |
| 3. Nervous System, . . . . . . . | 182 | 103 | 112 | 120 | 63 | 61 | 62 | 40 | 51 | 397 |
| 4. Organs of Respiration, . . . . | 329 | 200 | 132 | 177 | 105 | 62 | 152 | 95 | 70 | 661 |
| 5. " Circulation, . . . . | 27 | 17 | 24 | 19 | 11 | 11 | 8 | 6 | 13 | 68 |
| 6. " Digestion, . . . . . | 61 | 33 | 40 | 30 | 13 | 24 | 31 | 20 | 16 | 134 |
| 7. Urinary Organs, . . . . . . . | 7 | 3 | 3 | 3 | 3 | 1 | 4 | | 2 | 13 |
| 8. Organs of Generation, . . . . | 4 | 4 | 9 | | | | 4 | 4 | 9 | 17 |
| 9. " Locomotion, . . . . | 4 | 2 | 6 | 2 | 1 | 3 | 2 | 1 | 3 | 12 |
| 10. Integumentary System, . . . . | 1 | 3 | 2 | 1 | 2 | 1 | | 1 | 1 | 6 |
| 11. Old Age, . . . . . . . . . . . | 12 | 18 | 10 | 4 | 6 | 6 | 8 | 12 | 4 | 40 |
| 12. External Causes, . . . . . . . | 31 | 31 | 42 | 28 | 23 | 31 | 3 | 8 | 11 | 104 |
| Still Born, . . . . . . . . . | 49 | 36 | 40 | 32 | 23 | 20 | 17 | 13 | 20 | 125 |
| Unknown, . . . . . . . . . . | 40 | 22 | 16 | 25 | 8 | 11 | 15 | 14 | 5 | 78 |
| | 1070 | 693 | 635 | 602 | 380 | 316 | 468 | 313 | 319 | 2398 |

TABLE No. 2.

1. *Endemic and Contagious Diseases—Zymotic or Epidemic.*

| | Males. | Females. | Boys. | Girls. | Under 1 yr. | 1 to 2. | 2 to 5. | 5 to 10. | 10 to 15. | 15 to 20. | 20 to 30. | 30 to 40. | 40 to 50. | 50 to 60. | 60 to 70. | 70 to 80. | 80 to 90. | 9t o 100. | 100 to 110. | Total. |
|---|---|---|---|---|---|---|---|---|---|---|---|---|---|---|---|---|---|---|---|---|
| Cholera, . . . . . | 2 | | | | | | | | | | | 1 | | 1 | | | | | | 2 |
| " Infantum, . | 3 | 7 | 3 | 7 | 8 | 2 | | | | | | | | | | | | | | 10 |
| " Morbus, . . | | 1 | | | | | | | | | | | | | | 1 | | | | 1 |
| Croup, . . . . . | 31 | 34 | 31 | 31 | 11 | 9 | 34 | 8 | | | | | 3 | | | | | | | 65 |
| Diarrhœa, . . . . | 12 | 6 | 6 | 3 | 3 | 5 | 1 | | | | 2 | 2 | 1 | 1 | 1 | 1 | 1 | | | 18 |
| Dysentery, . . . . | 10 | 8 | 4 | 5 | 3 | 5 | 1 | | | | 1 | 3 | | 2 | 1 | 1 | 1 | | | 18 |
| Erysipelas, . . . . | 12 | 16 | 5 | 10 | 8 | 1 | | 1 | 2 | 3 | 1 | 3 | 2 | 3 | 1 | 2 | 1 | | | 28 |
| Fever, Bilious, . . . | 3 | | 1 | | | | 1 | | | | 1 | 1 | | | | | | | | 3 |
| " Congestive, . | 1 | | 1 | | | | | | | 1 | | | | | | | | | | 1 |
| " Gastric, . . | | 1 | | 1 | 1 | | | | | | | | | | | | | | | 1 |
| " Enteritic, . . | 2 | | | | | | | | | | | | 1 | | 1 | | | | | 2 |
| " Inflammatory, | | 1 | | 1 | 1 | | | | | | | | | | | | | | | 1 |
| " Intermittent, . | | 2 | | 1 | | | 1 | | | | | | | | | 1 | | | | 2 |
| " Nervous, . . | | 1 | | | | | | | | | | | | | | 1 | | | | 1 |
| " Remittent,. . | 1 | 4 | 1 | 2 | 2 | | | 1 | | | 2 | | | | | | | | | 5 |
| " Scarlet, . . . | 72 | 81 | 72 | 80 | 11 | 31 | 66 | 36 | 7 | 1 | | | 1 | | | | | | | 153 |
| " Typhoid, . . | 17 | 19 | 6 | 12 | 2 | 1 | 4 | 6 | 2 | 3 | 3 | 5 | 3 | 3 | 2 | 2 | | | | 36 |
| " Typhus, . . | 2 | 3 | | 1 | | | 1 | | | | 1 | 1 | | | 1 | 1 | | | | 5 |
| Hooping-Cough, . . | 2 | 8 | 2 | 8 | 6 | 1 | 2 | 1 | | | | | | | | | | | | 10 |
| Measles, . . . . . . | 6 | 5 | 6 | 5 | 2 | 3 | 3 | 2 | 1 | | | | | | | | | | | 11 |
| Influenza, . . . . | 1 | 1 | 1 | | | | | 1 | | | | | | | | | 1 | | | 2 |
| Small-pox, . . . . | 12 | 14 | 11 | 11 | 6 | 9 | 5 | 1 | | 1 | 2 | 2 | | | | | | | | 26 |
| Syphilis, . . . . . | | 1 | | 1 | 1 | | | | | | | | | | | | | | | 1 |
| Varioloid, . . . . | 3 | | 3 | | 1 | | 1 | 1 | | | | | | | | | | | | ? |
| | 192 | 213 | 153 | 179 | 66 | 67 | 120 | 58 | 12 | | 13 | 18 | 11 | 10 | 7 | 10 | 4 | | | 405 |

2. *Uncertain or General Seat—Sporadic Diseases.*

| | Male. | Female. | Boys. | Girls. | Under 1 yr. | 1 to 2. | 2 to 5. | 5 to 10. | 10 to 15. | 15 to 20. | 20 to 30. | 30 to 40. | 40 to 50. | 50 to 60. | 60 to 70. | 70 to 80. | 80 to 90. | 90 to 100. | 100 to 110. | Total. |
|---|---|---|---|---|---|---|---|---|---|---|---|---|---|---|---|---|---|---|---|---|
| Abscess, . . . . . . | 9 | 4 | 3 | 1 | 3 | | | | | 1 | 2 | 2 | 3 | 1 | | 1 | | | | 13 |
| Congestion, . . . . . | 1 | 1 | 1 | 1 | 1 | 1 | | | | | | | | | | | | | | 2 |
| Cachexia, . . . . . . | 1 | | | | | | | | | | | | | | 1 | | | | | 1 |
| Cancer, . . . . . . . | 6 | 16 | | 2 | | 1 | | | 1 | | 1 | 4 | 4 | 4 | 3 | 4 | | | | 22 |
| Cyanosis, . . . . . . | 1 | 6 | 1 | 6 | 7 | | | | | | | | | | | | | | | 7 |
| Debility, . . . . . . . | 46 | 29 | 20 | 19 | 34 | 3 | 1 | 1 | | | 1 | 3 | 2 | 9 | 11 | 6 | 4 | | | 75 |
| Disease of Throat, . . | 1 | | 1 | | 1 | | | | | | | | | | | | | | | 1 |
| Dropsy, . . . . . . . | 28 | 29 | 13 | 10 | 2 | 2 | 7 | 10 | 1 | 1 | 9 | 3 | 5 | 4 | 6 | 4 | 3 | | | 57 |
| Gangrene, . . . . . . | 4 | 2 | 2 | | 1 | | | | | 1 | | | | 1 | 1 | 1 | | 1 | | 6 |
| Gout, . . . . . . . . | | 2 | | | | | | | | | | | 1 | | | | 1 | | | 2 |
| Hemorrhage, . . . . . | 2 | 2 | | 1 | 1 | | | | | | 1 | 2 | | | | | | | | 4 |
| Inanition, . . . . . . | 13 | 6 | 12 | 5 | 14 | | 2 | | 1 | | | | | 1 | | 1 | | | | 19 |
| Inflammation, . . . . . | 1 | 2 | 1 | 2 | | 1 | | 1 | 1 | | | | | | | | | | | 3 |
| " Throat, . | 1 | 1 | 1 | 1 | | 2 | | | | | | | | | | | | | | 2 |
| Malformation, . . . . . | 2 | 3 | 2 | 3 | 5 | | | | | | | | | | | | | | | 5 |
| Mortification, . . . . . | 3 | 3 | 2 | | | | | 2 | | | | | | | 1 | 2 | 1 | | | 6 |
| Marasmus, . . . . . . | 47 | 39 | 42 | 35 | 49 | 13 | 12 | 3 | | | 1 | | 2 | 2 | 1 | 2 | | 1 | | 86 |
| Rickets, . . . . . . . | 1 | | 1 | | | 1 | | | | | | | | | | | | | | 1 |
| Scrofula, . . . . . . | 6 | 4 | 5 | 3 | 2 | 3 | 1 | | | 2 | 1 | 1 | | | | | | | | 10 |
| Sore Throat, . . . . . | 2 | 5 | 2 | 5 | 1 | 4 | | 2 | | | | | | | | | | | | 7 |
| Tabes Mesenterica, . . | | 4 | | 4 | | 1 | 3 | | | | | | | | | | | | | 4 |
| Tumor, . . . . . . . | 1 | 2 | 1 | | | | | 1 | | | | 1 | 1 | | | | | | | 3 |
| " on Neck, . . | | 1 | | 1 | 1 | | | | | | | | | | | | | | | 1 |
| Ulceration, . . . . . . | | 1 | | | | | | | | | | 1 | | | | | | | | 1 |
| | 176 | 162 | 110 | 99 | 122 | 32 | 26 | 20 | 4 | 5 | 16 | 17 | 18 | 22 | 24 | 21 | 9 | 2 | | 338 |

3. *Nervous Diseases.*

| | Male. | Female. | Boys. | Girls. | Under 1 yr. | 1 to 2. | 2 to 5. | 5 to 10. | 10 to 15. | 15 to 20. | 20 to 30. | 30 to 40. | 40 to 50. | 50 to 60. | 60 to 70. | 70 to 80. | 80 to 90. | 90 to 100. | 100 to 110. | Total. |
|---|---|---|---|---|---|---|---|---|---|---|---|---|---|---|---|---|---|---|---|---|
| Cramp, . . . . . . . | | 1 | | 1 | | | 1 | | | | | | | | | | | | | 1 |
| Apoplexy, . . . . . . | 20 | 13 | 5 | 2 | 4 | | 2 | 1 | | | 1 | 2 | 2 | 8 | 4 | 6 | 3 | | | 33 |
| Chorea, . . . . . . . | | 1 | | | | | | | | | 1 | | | | | | | | | 1 |
| Concussion of Brain, . | 3 | | | | | | | | | | | 1 | 1 | | | 1 | | | | 3 |
| Congestion of Brain, . | 31 | 13 | 21 | 9 | 15 | 4 | 8 | 3 | | | 1 | 3 | 3 | 3 | 2 | 1 | 1 | | | 44 |
| Convulsions, . . . . . | 65 | 56 | 62 | 51 | 68 | 14 | 15 | 11 | 1 | 4 | 4 | 2 | | 2 | | | | | | 121 |
| Disease of Brain, . . | 12 | 3 | 8 | 2 | 3 | 5 | 1 | | | 1 | | 1 | 1 | 1 | 2 | | | | | 15 |
| " Ear, . . . | 1 | | 1 | | | 1 | | | | | | | | | | | | | | 1 |
| Dropsy of Brain, . . . | 25 | 15 | 23 | 14 | 10 | 16 | 9 | 2 | | | | 2 | | | | | 1 | | | 40 |
| Effusion on Brain, . . | 9 | 4 | 6 | 4 | 4 | 3 | 2 | 1 | | | | 1 | | 1 | 1 | | | | | 13 |
| Epilepsy, . . . . . . | 2 | | 1 | | | | 1 | | | | | | 1 | | | | | | | 2 |
| Inflammation of Brain, | 34 | 28 | 25 | 23 | 13 | 8 | 14 | 8 | 3 | 2 | 4 | 5 | 2 | 3 | | | | | | 62 |
| Mania, . . . . . . . | 2 | 2 | | | | | | | | | 2 | 1 | | | | 1 | | | | 4 |
| Mania-a-Potu, . . . | 15 | 4 | | | | | | | | | 2 | 9 | 6 | 1 | | 1 | | | | 19 |
| Palsy, . . . . . . . | 17 | 9 | | | | | | | | | 2 | 1 | 5 | 4 | 7 | 6 | 1 | | | 26 |
| Softening of Brain, . . | 5 | 2 | 3 | 1 | 1 | 1 | 1 | | 1 | | | | 1 | 1 | 1 | | | | | 7 |
| Tetanus, . . . . . . | 3 | 2 | 2 | | | 1 | | | 1 | | 2 | 1 | | | | | | | | 5 |
| | 244 | 153 | 157 | 107 | 118 | 53 | 54 | 26 | 6 | 7 | 19 | 29 | 22 | 24 | 17 | 16 | 6 | | | 397 |

4. *Organs of Respiration.*

| | Male. | Female. | Boys. | Girls. | Under 1 yr. | 1 to 2. | 2 to 5. | 5 to 10. | 10 to 15. | 15 to 20. | 20 to 30. | 30 to 40. | 40 to 50. | 50 to 60. | 60 to 70. | 70 to 80. | 80 to 90. | 90 to 100. | 100 to 110. | Total. |
|---|---|---|---|---|---|---|---|---|---|---|---|---|---|---|---|---|---|---|---|---|
| Asthma, . . . . . . | 1 | 3 | | 1 | | | | | 1 | | | 1 | 1 | | | 1 | | | | 4 |
| Congestion of Lungs, . | 16 | 13 | 10 | 9 | 9 | 2 | 7 | | 1 | | 3 | 2 | 3 | 1 | 1 | | | | | 29 |
| Consumption of Lungs, | 179 | 180 | 25 | 32 | 9 | 7 | 12 | 1 | 3 | 25 | 108 | 86 | 48 | 37 | 12 | 8 | 3 | | | 359 |
| Coryza, . . . . . . | | 1 | | 1 | 1 | | | | | | | | | | | | | | | 1 |
| Disease of Chest, . . | 1 | 1 | 1 | 1 | | 1 | 1 | | | | | | | | | | | | | 2 |
| " Lungs, . . | 10 | 4 | 5 | 1 | | 4 | 1 | | | 1 | 1 | 2 | 3 | | 1 | 1 | | | | 14 |
| Dropsy of Chest, . . | 12 | 7 | 7 | 3 | 4 | 3 | 3 | | | | | | | 3 | 3 | 1 | 2 | | | 19 |
| Effusion on Chest, . . | 2 | | 1 | | 1 | | | | | | | | 1 | | | | | | | 2 |
| Empyema, . . . . . . | 1 | | 1 | | | | 1 | | | | | | | | | | | | | 1 |
| Emphysema, . . . . . | 1 | | 1 | | 1 | | | | | | | | | | | | | | | 1 |
| Gangrene of Lungs, . | | 1 | | | | | | | | | | | | 1 | | | | | | 1 |
| Hectic Fever, . . . . | | 1 | | | | | | | | | 1 | | | | | | | | | 1 |
| Hemr'ge from Lungs, . | 6 | 1 | 1 | | 1 | | | | | | | 1 | 1 | 1 | 1 | 1 | 1 | | | 7 |
| Inflam'n of Bronchi, . | 28 | 25 | 20 | 19 | 17 | 8 | 6 | 7 | | 1 | 2 | 3 | 2 | 3 | 1 | 2 | 1 | | | 53 |
| " Lungs, . | 70 | 65 | 45 | 38 | 34 | 15 | 20 | 5 | 3 | 6 | 11 | 5 | 6 | 9 | 9 | 9 | 3 | | | 135 |
| " Larynx, . | 2 | 1 | 2 | 1 | 2 | | 1 | | | | | | | | | | | | | 3 |
| " Chest, . . | 3 | 2 | 3 | 1 | 2 | 1 | 1 | | | | | | | | 1 | | | | | 5 |
| " Pleura, . | 8 | 9 | 1 | 2 | 1 | | | 1 | | 1 | 2 | 3 | 2 | 4 | 1 | 2 | | | | 17 |
| " Trachea, . | 4 | 1 | 4 | 1 | | 2 | 2 | 1 | | | | | | | | | | | | 5 |
| " Tonsils, . | | 2 | | 2 | 1 | | | 1 | | | | | | | | | | | | 2 |
| | 344 | 317 | 127 | 112 | 83 | 43 | 55 | 16 | 8 | 34 | 128 | 103 | 67 | 59 | 30 | 25 | 10 | | | 661 |

5. *Organs of Circulation.*

| | Males. | Females. | Boys. | Girls. | Und'r 1 yr. | 1 to 2. | 2 to 5. | 5 to 10. | 10 to 15. | 15 to 20. | 20 to 30. | 30 to 40. | 40 to 50. | 50 to 60. | 60 to 70. | 70 to 80. | 80 to 90. | 90 to 100. | 100 to 110. | Total. |
|---|---|---|---|---|---|---|---|---|---|---|---|---|---|---|---|---|---|---|---|---|
| Aneurism, . . . . . | 2 | | 1 | | 1 | | | | | | | | | | | 1 | | | | 2 |
| Disease of Heart, . . . | 27 | 20 | 11 | 4 | 3 | 1 | 3 | 2 | 4 | 2 | 6 | 4 | 6 | 1 | 12 | 3 | | | | 47 |
| Dropsy of Heart, . . . | 4 | 3 | 2 | 1 | 1 | | | 1 | 1 | | 1 | | 1 | | | 2 | | | | 7 |
| Enlargement of Heart, . | 4 | 4 | 1 | | | | | | 1 | | 1 | 1 | 1 | 2 | 1 | 1 | | | | 8 |
| Inflammation of " . | 3 | | 2 | | | | 2 | | | | | | 1 | | | | | | | 3 |
| Rupture of Bloodvessel, | 1 | | | | | | | | | | | 1 | | | | | | | | 1 |
| | 41 | 27 | 17 | 5 | 5 | 1 | 5 | 3 | 6 | 2 | 8 | 6 | 9 | 3 | 13 | 7 | | | | 68 |

6. *Digestive Organs.*

| | Male. | Female. | Boys. | Girls. | Under 1 yr. | 1 to 2. | 2 to 5. | 5 to 10. | 10 to 15. | 15 to 20. | 20 to 30. | 30 to 40. | 40 to 50. | 50 to 60. | 60 to 70. | 70 to 80. | 80 to 90. | 90 to 100. | 100 to 110. | Total. |
|---|---|---|---|---|---|---|---|---|---|---|---|---|---|---|---|---|---|---|---|---|
| Cancrum Oris, . . . . | | 1 | | 1 | | | 1 | | | | | | | | | | | | | 1 |
| Cirrhosis, . . . . . . | 1 | | | | | | | | | | | | | | 1 | | | | | 1 |
| Congestion of Bowels, . | 1 | 1 | | | | | | | | | | 2 | | | | | | | | 2 |
| Colic, . . . . . . . | | 2 | | 1 | 1 | | | | | | | | | 1 | | | | | | 2 |
| Disease of Liver, . . . | 5 | 1 | 3 | | 2 | | 1 | | | | 1 | 1 | | | 1 | | | | | 6 |
| " Stom. & Bow., | 2 | 4 | 1 | 2 | 1 | | 1 | 1 | | | | 1 | | | | 2 | | | | 6 |
| Dropsy Abdominal, . . | 2 | | | | | | | | | | | | | 1 | 1 | | | | | 2 |
| Dyspepsia, . . . . . . | | 1 | | | | | | | | | | | 1 | | | | | | | 1 |
| Hernia, Strangulated, . | 1 | 2 | | | | | | | | | | 1 | 1 | 1 | | | | | | 3 |
| Jaundice, . . . . . . | 3 | 2 | 3 | 1 | 4 | | | | | | | | | | 1 | | | | | 5 |
| Inflamm'n of Abdomen, . | | 1 | | 1 | | 1 | | | | | | | | | | | | | | 1 |
| " Liver, . . . | 6 | 5 | 2 | 3 | 1 | | | 2 | 1 | 1 | 1 | | 3 | 2 | | | | | | 11 |
| " Peritoneum, . | 5 | 14 | | 6 | 1 | 1 | | 2 | | 2 | 5 | 3 | 1 | 2 | 1 | 1 | | | | 19 |
| " Stom. and Bow., | 35 | 30 | 18 | 11 | 11 | 3 | 7 | 3 | 1 | 4 | 3 | 12 | 5 | 3 | 8 | 3 | 2 | | | 65 |
| Intussusception, . . . | 1 | | | | | | | | | | | | | | 1 | | | | | 1 |
| Obstruction of Bowels, . | 1 | | 1 | | 1 | | | | | | | | | | | | | | | 1 |
| Scirrhus of Stomach, . | 1 | | | | | | | | | | | | 1 | | | | | | | 1 |
| Sore Mouth, . . . . | | 1 | | 1 | | 1 | | | | | | | | | | | | | | 1 |
| Teething, . . . . . . | 2 | 1 | 2 | 1 | | 3 | | | | | | | | | | | | | | 3 |
| Ulceration of Stomach, . | 1 | | | | | | | | | | | | | 1 | | | | | | 1 |
| Worms, . . . . . . . | | 1 | | 1 | | 1 | | | | | | | | | | | | | | 1 |
| | 67 | 67 | 30 | 29 | 22 | 10 | 10 | 8 | 2 | 7 | 10 | 20 | 12 | 11 | 14 | 6 | 2 | | | 134 |

7. *Urinary Organs.*

| | Male. | Female. | Boys. | Girls. | Under 1 yr. | 1 to 2. | 2 to 5. | 5 to 10. | 10 to 15. | 15 to 20. | 20 to 30. | 30 to 40. | 40 to 50. | 50 to 60. | 60 to 70. | 70 to 80. | 80 to 90. | 90 to 100. | 100 to 110. | Total. |
|---|---|---|---|---|---|---|---|---|---|---|---|---|---|---|---|---|---|---|---|---|
| Albuminuria, . . . . | 4 | | | | | | | | | | | 2 | 1 | 1 | | | | | | 4 |
| Diabetes, . . . . . | | 2 | | | | | | | | | | 1 | 1 | | | | | | | 2 |
| Disease of Kidneys, . . | 1 | 1 | | 1 | | | | | 1 | | | 1 | | | | | | | | 2 |
| Inflamm'n of Bladder, . | | 1 | | | | | | | | | | | | | | 1 | | | | 1 |
| " Kidneys, . . | 2 | 1 | 1 | | | | | 1 | | | 1 | | 1 | | | | | | | 3 |
| Suppression of Urine, . | | 1 | | 1 | | | 1 | | | | | | | | | | | | | 1 |
| | 7 | 6 | 1 | 2 | | | 1 | 1 | 1 | | 1 | 4 | 3 | 1 | | 1 | | | | 13 |

8. *Organs of Generation.*

| | Male. | Female. | Boys. | Girls. | Under 1 yr. | 1 to 2. | 2 to 5. | 5 to 10. | 10 to 15. | 15 to 20. | 20 to 30. | 30 to 40. | 40 to 50. | 50 to 60. | 60 to 70. | 70 to 80. | 80 to 90. | 90 to 100. | 100 to 110. | Total. |
|---|---|---|---|---|---|---|---|---|---|---|---|---|---|---|---|---|---|---|---|---|
| Fever, Puerperal, . . . | | 12 | | 2 | | | | | | 2 | 3 | 6 | 1 | | | | | | | 12 |
| Hem'age from Uterus, . | | 2 | | 1 | | | 1 | | | | 1 | | | | | | | | | 2 |
| Inflamm'n of Uterus, . . | | 2 | | | | | | | | | 1 | 1 | | | | | | | | 2 |
| Ovarian Tumor, . . . | | 1 | | | | | | | | | | | 1 | | | | | | | 1 |
| | | 17 | | 3 | | | 1 | | | 2 | 5 | 7 | 2 | | | | | | | 17 |

9. *Organs of Locomotion.*

| | Male. | Female. | Boys. | Girls. | Under 1 yr. | 1 to 2. | 2 to 5. | 5 to 10. | 10 to 15. | 15 to 20. | 20 to 30. | 30 to 40. | 40 to 50. | 50 to 60. | 60 to 70. | 70 to 80. | 80 to 90. | 90 to 100. | 100 to 110. | Total. |
|---|---|---|---|---|---|---|---|---|---|---|---|---|---|---|---|---|---|---|---|---|
| Caries, . . . . . . . | 1 | | 1 | | | | 1 | | | | | | | | | | | | | 1 |
| Caries of Vertebræ, . . | | 1 | | | | | | | | | | 1 | | | | | | | | 1 |
| Disease of Hip, . . . | | 1 | | | | | | | | | 1 | | | | | | | | | 1 |
| " Spine, . . . | 1 | | | | | | | | | | | | 1 | | | | | | | 1 |
| Inflamm'n of Hip, . . . | | 1 | | 1 | | | | | 1 | | | | | | | | | | | 1 |
| " Spine, . . | 2 | | 1 | | 1 | | | | | | | | 1 | | | | | | | 2 |
| Effusion Spinal Marrow, | | 1 | | | | | | | | | | | | | 1 | | | | | 1 |
| Rheumatism, . . . . | 2 | 2 | 2 | | 1 | | 1 | | | | 1 | | | | 1 | | | | | 4 |
| | 6 | 6 | 4 | 1 | 2 | | 2 | | 1 | | 2 | 1 | 2 | | 2 | | | | | 12 |

10. *Integumentary System.*

| | Male. | Female. | Boys. | Girls. | Under 1 yr. | 1 to 2. | 2 to 5. | 5 to 10. | 10 to 15. | 15 to 20. | 20 to 30. | 30 to 40. | 40 to 50. | 50 to 60. | 60 to 70. | 70 to 80. | 80 to 90. | 90 to 100. | 100 to 110. | Total. |
|---|---|---|---|---|---|---|---|---|---|---|---|---|---|---|---|---|---|---|---|---|
| Eczema, . . . . . . . | | 1 | | 1 | 1 | | | | | | | | | | | | | | | 1 |
| Purpura, . . . . . . | 1 | | 1 | | | | | 1 | | | | | | | | | | | | 1 |
| Scurvy, . . . . . . . | 3 | 1 | | | | | | | | | | 1 | 2 | | 1 | | | | | 4 |
| | 4 | 2 | 1 | 1 | 1 | | | 1 | | | | 1 | 2 | | 1 | | | | | 6 |

11. *Old Age.*

| | | | | | | | | | | | | | | | | | | | | |
|---|---|---|---|---|---|---|---|---|---|---|---|---|---|---|---|---|---|---|---|---|
| Old Age, . . . . . . . | 16 | 24 | | | | | | | | | | | | | 2 | 7 | 23 | 6 | 2 | 40 |

12. *External Causes.*

| | Male. | Female. | Boys. | Girls. | Under 1 yr. | 1 to 2. | 2 to 5. | 5 to 10. | 10 to 15. | 15 to 20. | 20 to 30. | 30 to 40. | 40 to 50. | 50 to 60. | 60 to 70. | 70 to 80. | 80 to 90. | 90 to 100. | 100 to 110. | Total. |
|---|---|---|---|---|---|---|---|---|---|---|---|---|---|---|---|---|---|---|---|---|
| Asphyxia, . . . . . | 2 | 1 | 2 | 1 | 3 | | | | | | | | | | | | | | | 3 |
| Burns, . . . . . . . | 8 | 1 | 5 | 1 | | 2 | 3 | | 1 | | 2 | 1 | | | | | | | | 9 |
| Casualties, . . . . . | 16 | 3 | 2 | 1 | | | 1 | 2 | | | 6 | 3 | 4 | 2 | 1 | | | | | 19 |
| Drowned, . . . . . . | 40 | 4 | 18 | 2 | 2 | | 2 | 8 | 4 | 4 | 6 | 6 | 5 | 5 | 2 | | | | | 44 |
| Exhaustion, . . . . . | 2 | | | | | | | | | | | | | | | 1 | 1 | | | 2 |
| Infanticide, . . . . . | 1 | | 1 | | 1 | | | | | | | | | | | | | | | 1 |
| Intemperance, . . . . | 3 | 4 | | | | | | | | | 1 | 3 | 1 | 1 | 1 | | | | | 7 |
| Poisoning, . . . . . . | | 2 | | | | | | | | | 1 | 1 | | | | | | | | 2 |
| Strangulation, . . . . | 1 | | | | | | | | | | | | 1 | | | | | | | 1 |
| Suffocation, . . . . . | | 4 | | 4 | 3 | | | 1 | | | | | | | | | | | | 4 |
| Suicide, . . . . . . . | 6 | 3 | | | | | | | | | 1 | 5 | 1 | 2 | | | | | | 9 |
| Violence, . . . . . . | 3 | | | | | | | | | | 2 | | 1 | | | | | | | 3 |
| | 82 | 22 | 28 | 9 | 9 | 2 | 6 | 11 | 5 | 4 | 19 | 19 | 13 | 10 | 4 | 1 | 1 | | | 104 |

| | Male. | Female. | Boys. | Girls. | Under 1 yr. | 1 to 2. | 2 to 5. | 5 to 10. | 10 to 15. | 15 to 20. | 20 to 30. | 30 to 40. | 40 to 50. | 50 to 60. | 60 to 70. | 70 to 80. | 80 to 90. | 90 to 100. | 100 to 110. | Total. |
|---|---|---|---|---|---|---|---|---|---|---|---|---|---|---|---|---|---|---|---|---|
| Stillborn, . . . . | 75 | 50 | 75 | 50 | 125 | | | | | | | | | | | | | | | 125 |
| Unknown, . . . . | 44 | 34 | 14 | 15 | 21 | 1 | 3 | 3 | | 1 | 4 | 5 | 20 | 9 | 7 | 3 | 1 | | | 78 |

TABLE No. 3.

*Deaths for the Second Quarter of* 1857, *at fifteen distinct periods of life.*

| | | | |
|---|---|---|---|
| Under 1 year, . . . . . . . . . | 449 | 60 to 70, . . . . . . . . | 121 |
| 1 to 2, . . . . . . . . . | 209 | 70 to 80, . . . . . . . . | 97 |
| 2 to 5, . . . . . . . . . | 283 | 80 to 90, . . . . . . . . | 56 |
| 5 to 10, . . . . . . . . | 147 | 90 to 100, . . . . . . . . | 8 |
| 10 to 15, . . . . . . . . | 45 | 100 to 110, . . . . . . . . | 2 |
| 15 to 20, . . . . . . . . | 71 | | |
| 20 to 30, . . . . . . . . | 225 | | 2,273 |
| 30 to 40, . . . . . . . . | 230 | Stillborn, . . . . | 125 |
| 40 to 50, . . . . . . . . | 181 | | |
| 50 to 60, . . . . . . . . | 149 | Total, . . . . . . | 2,398 |

Of the above deaths, 170 were from the Blockley Almshouse; 147 from the colored population; and 19 from the country, as follows:

| | April. | May. | June. | Total. |
|---|---|---|---|---|
| Almshouse, . . . | 72 | 53 | 45 | 170 |
| Blacks, . . . . . | 56 | 43 | 48 | 147 |
| Country, . . . . | 7 | 4 | 8 | 19 |
| | 135 | 100 | 101 | 336 |

TABLE No. 4.

The following table furnishes the number of deaths for each week during the quarter, together with the sexes, adults and children.

| | Male. | Female. | Boys. | Girls. | Adults. | Children. | Total. | Monthly Totals. |
|---|---|---|---|---|---|---|---|---|
| 1857. From Mar. 28th to Ap. 4th, | 114 | 99 | 65 | 59 | 89 | 124 | 213 | |
| " Ap. 4th to 11th, . . | 122 | 100 | 80 | 59 | 83 | 139 | 222 | |
| " Ap. 11th to 18th, . . | 135 | 91 | 74 | 49 | 103 | 123 | 226 | |
| " Ap. 18th to 25th, . . | 123 | 93 | 66 | 61 | 89 | 127 | 216 | |
| " Ap. 25th to May 2d, . | 109 | 84 | 53 | 48 | 92 | 101 | 193 | 1070 |
| " May 2d to May 9th, . | 105 | 79 | 62 | 41 | 81 | 103 | 184 | |
| " May 9th to 16th, . . | 84 | 88 | 51 | 45 | 76 | 96 | 172 | |
| " May 16th to 23d, . . | 105 | 78 | 57 | 38 | 88 | 95 | 183 | |
| " May 23d to 30th, . . | 85 | 69 | 46 | 41 | 67 | 87 | 154 | 693 |
| " May 30th to June 6th, | 92 | 82 | 56 | 46 | 72 | 102 | 174 | |
| " June 6th to 13th, . . | 69 | 84 | 34 | 47 | 72 | 81 | 153 | |
| " June 13th to 20th, . | 86 | 91 | 38 | 48 | 91 | 86 | 177 | |
| " June 20th to 27th, . | 69 | 62 | 34 | 31 | 66 | 65 | 131 | 635 |
| | 1298 | 1100 | 716 | 613 | 1069 | 1329 | 2398 | 2398 |

